# Separation increases passive stress-coping behaviors during forced swim and alters hippocampal dendritic morphology in California mice

**DOI:** 10.1371/journal.pone.0175713

**Published:** 2017-04-13

**Authors:** Molly M. Hyer, Erica R. Glasper

**Affiliations:** 1 Program in Neuroscience and Cognitive Sciences, University of Maryland, College Park, Maryland, United States of America; 2 Department of Psychology, University of Maryland, College Park, Maryland, United States of America; Universite de Rennes 1, FRANCE

## Abstract

Individuals within monogamous species form bonds that may buffer against the negative effects of stress on physiology and behavior. In some species, involuntary termination of the mother-offspring bond results in increased symptoms of negative affect in the mother, suggesting that the parent-offspring bond may be equally as important as the pair bond. To our knowledge, the extent to which affect in paternal rodents is altered by involuntary termination of the father-offspring bond is currently unknown. Here, we investigated to what extent separation and paternal experience alters passive stress-coping behaviors and dendritic morphology in hippocampal subfields of California mice (*Peromyscus californicus*). Irrespective of paternal experience, separated mice displayed shorter latencies to the first bout of immobility, longer durations of immobility, and more bouts of immobility than control (non-separated) mice. This effect of separation was exacerbated by paternal experience in some measures of behavioral despair—separation from offspring further decreased the latency to immobility and increased bouts of immobility. In the dentate gyrus, separation reduced dendritic spine density regardless of paternal experience. Increased spine density was observed on CA1 basal, but not apical, dendrites following paternal experience. Regardless of offspring presence, fatherhood was associated with reduced apical dendritic spine density in area CA3 of the hippocampus. Separation enhanced complexity of both basal and apical dendrites in CA1, while fatherhood reduced dendritic complexity in this region. Our data suggest that forced dissolution of the pair bond induces passive stress-coping behaviors and contributes to region-specific alterations in hippocampal structure in California mouse males.

## Introduction

The formation of a pair bond is one of the strongest relationships observed between two individuals [1]. As seen in monogamous species, bonds are typically formed after individuals spend significant periods of time in physical contact with one another [2,3]. These bonds can serve as a social buffer [1], one that may protect from the deleterious effects of stress on physiology and behavior [4]. Given this, involuntary termination of social bonds may result in impairments to health, brain structure, or behavior—as has been demonstrated in rodents and non-human primates [5–8]. While bond formation and bond separation studies typically focus on interactions between adults [9], for many species, the parent-offspring bond is an equally important interaction—one that can have significant and long-lasting effects on offspring development [10] and the structure and function of the parental brain [11–14]. Much of our understanding of parent-offspring interactions results from observations of maternal rodents and offspring. While some male rodents display parenting behaviors [15, 16], which can result in parenting-induced alterations in brain plasticity [17], it is not known whether benefits of father-offspring interaction are long-lasting or the extent to which preventing this interaction negatively affects neural structure and/or affective behaviors in the paternal brain.

Prevailing evidence suggests that interactions with offspring can affect neuronal structure of the maternal brain in many regions, including the hippocampus and the prefrontal cortex. Dendritic spine density is increased in the dentate gyrus (DG; [12]) and area CA1 of the hippocampus of maternal rodents. Similar enhancements in dendritic spine density are observed in the prefrontal cortex [18]. However, maternal experience does not result in global enhancements in dendritic plasticity, as dendritic atrophy of area CA1 and CA3 of the hippocampus is observed following parturition in rats [19]. These effects of offspring on structural plasticity of the hippocampus may be driven by offspring contact alone, as virgin female rats exposed to pups, acutely or chronically, have enhanced neuroplasticity within the DG and subventricular zone (i.e., cell proliferation, cell survival; [20]), however, to our knowledge no studies have investigated whether pup exposure alters dendritic morphology in virgin females.

Similar to alterations in brain plasticity, emotionality in maternal rodents during the postpartum period can be impacted by interactions with offspring. In humans, mothers with increased positive feelings toward their baby had greater gray matter volume in areas of the brain associated with maternal motivation and behaviors [21]. Breastfeeding mothers report lower anxiety [22], while disrupted maternal care is linked to increased risk for postpartum depression and anxiety [23, 24]. These studies suggest that mother-infant contact may prevent development of negative affect during the postpartum period and studies in rodents appear to support this hypothesis. Repeated separation of rat mothers from offspring leads to increased depressive-like [25] and anxiety-like [26] behaviors in the dams. Reduced anxiety-like behavior in maternal rats is dependent on recent offspring contact [27]. Like alterations in structural morphology, exposure to pups alone can affect virgin females. Nulliparous female rats exposed to pups for 21 days show reduced depressive-like behavior on the forced swim task [28]. Taken together, the maternal literature suggests that the relationship between the mother and offspring is sufficient to permeate both the mother's behavior and neuronal morphology.

Due to the scarcity of mammalian models of paternal care, far less is known about how interactions with offspring alter the brain and behavior of fathers. Human fathers show enhanced brain activity in regions associated with parenting, like the ventral prefrontal cortex and amygdala, following exposure to infant cues [29]. Additionally, striatal volume is negatively correlated with symptoms of depression in fathers 12–16 weeks postpartum [30]. These findings in human fathers, while few, suggest that brain regions associated with parenting undergo plasticity during the postpartum period and may be sensitive to disruptions in normal parental care—an effect that is similar to observations in mothers. Affect and structural plasticity of the brain in rodent and non-human primate models of paternal care mimic what has been seen in rodent mothers. In the California mouse father, a reduction in anxiety-like behavior and a maintenance of adult-born neurons is observed on postnatal day (PND) 16 [12]—effects that may be driven by increased pup contact at this time [15]. Pup contact alone increases adult neurogenesis in the DG of male prairie voles (*Michrotus ochragaster*; [31]). Together, these data suggest that interaction with offspring influences both function and structure of the paternal brain.

Our goal was to enhance our knowledge of fatherhood-related neuroplasticity by determining to what extent early separation from offspring alters hippocampal dendritic morphology and affective behaviors during the forced swim test in fathers. We examined passive stress-coping strategies, during the forced swim test, followed by Golgi-Cox analysis of dendritic morphology in hippocampal subfields (DG, CA1, and CA3) of California mouse males that were separated from their offspring on PND 1, compared to males that remained with their offspring until weaning. Given that California mouse fathers play key roles in the physical and psychological development of their offspring [32, 33] and fatherhood alters emotional responsivity in this species [12, 34], we generated an a priori hypothesis that separation from offspring would be equally as important to the father's stress-coping strategies during the forced swim test compared to separation from the mate, as well as contribute to decreased dendritic plasticity across the hippocampus. The current data suggest that in California mouse fathers, pup exposure decreases passive coping strategies during the forced swim test and induces differential structural remodeling of the hippocampus. Taken together, these findings suggest that the father-offspring bond may be important for improving both affective behavior and structural plasticity in fathers of a biparental species.

## Materials and methods

### Animals

Virgin male, virgin female, and tubally ligated virgin female California mice (60–90 days of age) were obtained from the Peromyscus Genetic Stock Center (University of South Carolina, Columbia, SC) or were descendants of these mice bred in our colony. Mice were provided ad libitum access to food and water and were housed on a 16:8 reversed light/dark cycle (lights off at 10:00h). Males were pair housed (non-fathers, 37.33 ± 3.21 days; fathers, 58.95 ± 32.37 days) with gonadally intact or tubally ligated females, allowed to mate, and remained undisturbed throughout the duration of the pregnancy until PND 1 (where applicable). An average of 1.64 ± 0.58 offspring were in each litter. On PND 1, half of the males were removed from their home cages and individually housed, resulting in two groups [separated non-father (n = 10); separated father (n = 11)]. The remaining males were not disturbed until behavioral testing, resulting in two additional groups [control non-father (n = 8); control father (n = 12)]. All experiments were approved by the University of Maryland Institutional Animal Care and Use Committee and conformed to the guidelines provided by the National Institutes of Health for the care and use of animals.

A small subset of the data from these experiments, DG and CA1 dendritic morphology from control non-fathers and fathers, have been presented in a smaller study [34] that examined fatherhood-induced effects on dendritic morphology and anxiety-like behavior. Those data have been included in this article for adequate comparison to the separated groups. This is explicitly stated and referenced when these data are presented.

### Forced swim task

On PND 21 (time matched for non-fathers), all male mice were tested on the forced swim task, a common behavioral test used to assess active and passive stress-coping behavioral strategies [35]. The version of the task used here was modified to consist of a single session that is commonly used in mice, compared to 2 test sessions often used in rats [35, 36]. Testing began ~2hr after lights out and was performed under red light illumination. Males were placed in holding cages and transported to the behavioral room for testing. The forced swim test consisted of placing mice, for 5 minutes each, in a Plexiglas cylinder (30cm diameter, 43cm deep) that was filled ¾ of the way with 23–25°C tap water. Behavior was digitally recorded from a side view of the cylinder at 30 frames per second [37], to better distinguish between swimming and immobility behaviors. Behavior during the task was analyzed with EthoVision^®^XT 11 behavioral tracking software (Noldus, Leesburg, VA). The first 2 minutes of the task were used for habituation, while the final 3 minutes constituted the test portion of the task [38, 39]. Thus, the following behaviors were assessed from the latter 3 minutes of the task and used to assess passive stress-coping behavior: latency to the first bout of immobility, duration of immobility, and frequency of immobility bouts. Immobility was defined as remaining parallel to the surface of the water with only slight motions to remain afloat, while swimming was defined as continuous motion of paws and head. Use of automated detection systems typically reduces observer-related error. However, due to individual differences in behavioral patterns and minor changes in camera placement over the course of the experiment, slight modifications to the analysis parameters were made [37]. This resulted in different mobility settings for immobility when using EthoVision to analyze the behaviors. Immobility was set between 4% and 8% of pixel variation per 3 frames. The number of mice scored at each percentage of pixel variation are as follows: 4%, n = 1, 5%, n = 23, 7%, n = 1, 8%, n = 12. Flipping behavior during the forced swim task greatly increases the chance of drowning in California mice (unpublished observations); therefore, any mice that exhibited flipping behavior during the forced swim test were quickly removed and were not included in any analyses (n = 3). Following testing, mice were returned to their home cage and remained undisturbed until weaning of offspring on PND 35. Total duration outside of the home cage was <15 minutes.

### Golgi-impregnation

On PND 35, mice were euthanized via cervical dislocation and brains were harvested, rinsed with dH_2_O, and processed for Golgi impregnation per manufacturer’s recommendations (Rapid Golgi Staining Kit, FD Neurotechnologies, Columbia, MD). Briefly, brains were submerged into equal parts of solutions A and B and stored in the dark at room temperature. Solution was refreshed 24hrs later. Fourteen days later, brains were transferred to solution C and stored in the dark at -4°C for 24hrs. Solution C was refreshed and brains were maintained under these conditions for 10 days. Tissue, of 100μm thickness, was sectioned in solution C using a vibrating microtome (Leica Microsystems, Chicago, IL). Sections were immediately mounted onto gelatinized slides and allowed to dry overnight. Slides were then placed into mailers and rinsed with dH_2_O for 8min. Tissue was exposed to equal parts of solutions D and E for 10min, rinsed, and then dehydrated in increasing concentrations of ethanol (50%, 75%, 95%, and 100%), cleared in xylene, and coverslipped under Permount (Fisher Scientific, Fair Lawn, NJ).

### Dendritic remodeling analysis

Dendritic remodeling analyses were performed, as previously described [34]. Granule cells within the DG, as well as pyramidal cells in areas CA1 and CA3, were analyzed for spine density (100x under oil immersion), and dendritic length and number of branch points (40x under oil immersion). Analyses were conducted using a Zeiss AxioImager microscope with a stage controller and neuroimaging software (Neurolucida, Williston, VT) by trained individuals with no knowledge of groups. Dendritic spine density measurements were taken from dendritic sections, with a mean of 10.24μm. These sections were on dendritic branches that were 2.9 ± 0.83 branches from the soma. For all analyses, dendrites were fully stained, relatively isolated, and predominately in one focal plane. Five neurons per brain and five dendrites per neuron were analyzed. Neurons were sampled from the entire rostral-caudal extent of the hippocampus.

### Statistics

Data were analyzed using GraphPad Prism version 6.0f for Mac OS X, (GraphPad Software, La Jolla California USA, www.graphpad.com). All data were checked for equality of group variances and were transformed, if necessary, to meet normality assumptions. All behavioral data were log transformed [Y = Log (Y)]. Two-way analysis of variance (ANOVA) was used to assess the effects of separation and paternal experience on behavioral and neuronal endpoints. Main effects were considered statistically different when p ≤ 0.05. In cases where multiple comparisons were appropriate, a false discovery rate (FDR) analysis [40] was performed to correct for alpha inflation (desired false discovery rate (q) value was set to 1%). The unadjusted and adjusted P values (q values) are reported. Effect sizes were calculated for all neural plasticity analyses using Cohen’s *d* and eta squared for ANOVA. Statistical outliers were determined using the ROUT method that identifies outliers from a non-linear regression, which resulted in the exclusion of one separated control and one separated father from all analyses. Given all exclusions, the final sample sizes are as follows: control non-father, n = 8; control father, n = 12, separated non-father, n = 6; separated father n = 8.

## Results

### Behavior

**Fig 1:**
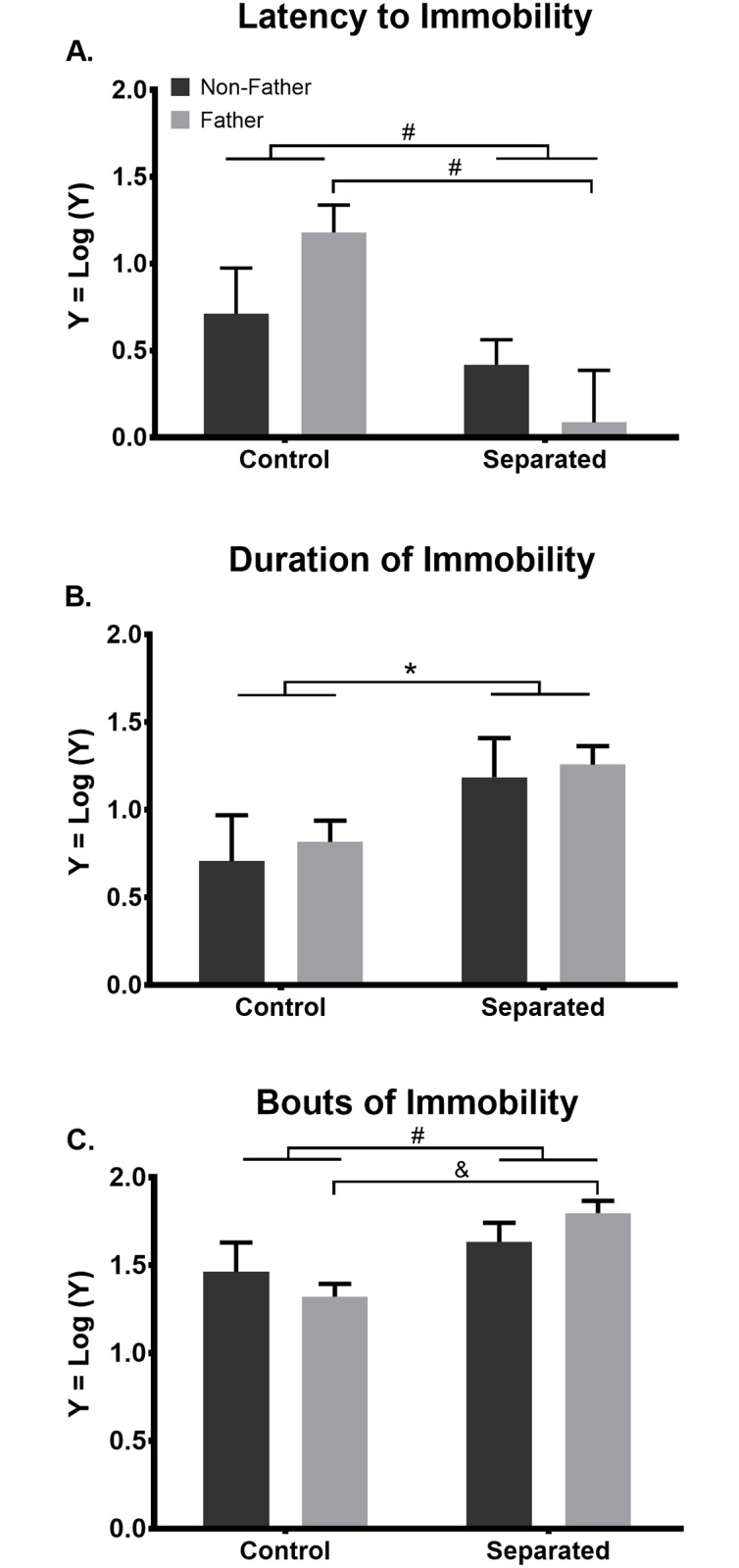
Separation increases passive stress-coping behavior during the forced swim task in male California mice. (A) Twenty days of separation, regardless of paternal experience, significantly shortens the latency to the initial bout of immobility during the forced swim task, compared to non-separated controls. Among paternal mice, separation from offspring exacerbates this effect, as separated fathers have a shorter latency to immobility than non-separated fathers. (B) Regardless of paternal experience, 20 days of separation increases the duration of immobility during the forced swim task. (C) Twenty days of separation, regardless of paternal experience, increases bouts of immobility during the forced swim task, compared to non-separated controls. Among paternal mice, separation from offspring exacerbates this effect as separated fathers have more bouts of immobility than non-separated fathers. *p ≤ 0.05, ^#^p ≤ 0.01, ^&^p ≤ 0.001. Bars represent mean + SEM.

Separation increased all measures of passive stress-coping behavior during the forced swim task. Latency to immobility was significantly altered by separation (F_(1, 30)_ = 9.019; p = 0.005) but not by paternal experience (p = 0.772). Overall, separation reduced the latency to become immobile (Fig 1A). No significant interaction between separation and paternal experience was observed (p = 0.095). Given our a priori hypothesis that the effects of separation would be greater in fathers compared to non-fathers, post-hoc analysis revealed that latency to immobility was significantly shorter in separated fathers than control fathers (p = 0.006; Fig 1A). This difference remained statistically significant after the FDR correction was applied (q = 0.0009). Non-separated, control and separated non-fathers did not significantly differ in their latency to immobility (p = 0.651). Duration of immobility was significantly increased by separation (F_(1,30)_ = 6.435; p = 0.017; Fig 1B) but not by paternal experience (p = 0.620). No interaction between separation and paternal experience was observed (p = 0.919). Bouts of immobility were similarly increased by separation (F_(1,30)_ = 0.683; p = 0.006) but not by paternal experience (p = 0.922). No interaction between separation and paternal experience was observed (p = 0.186). Given our a priori hypothesis that the effects of separation would be greater in fathers than non-fathers, post-hoc analysis revealed that more bouts of immobility were observed in separated fathers, compared to control fathers (p = 0.0128; Fig 1C). This difference remained significantly different following FDR correction (q = 0.000). Control and separated non-fathers did not significantly differ in bouts of immobility (p > 0.999).

### Dendritic morphology

#### Dendritic spine density

**Fig 2:**
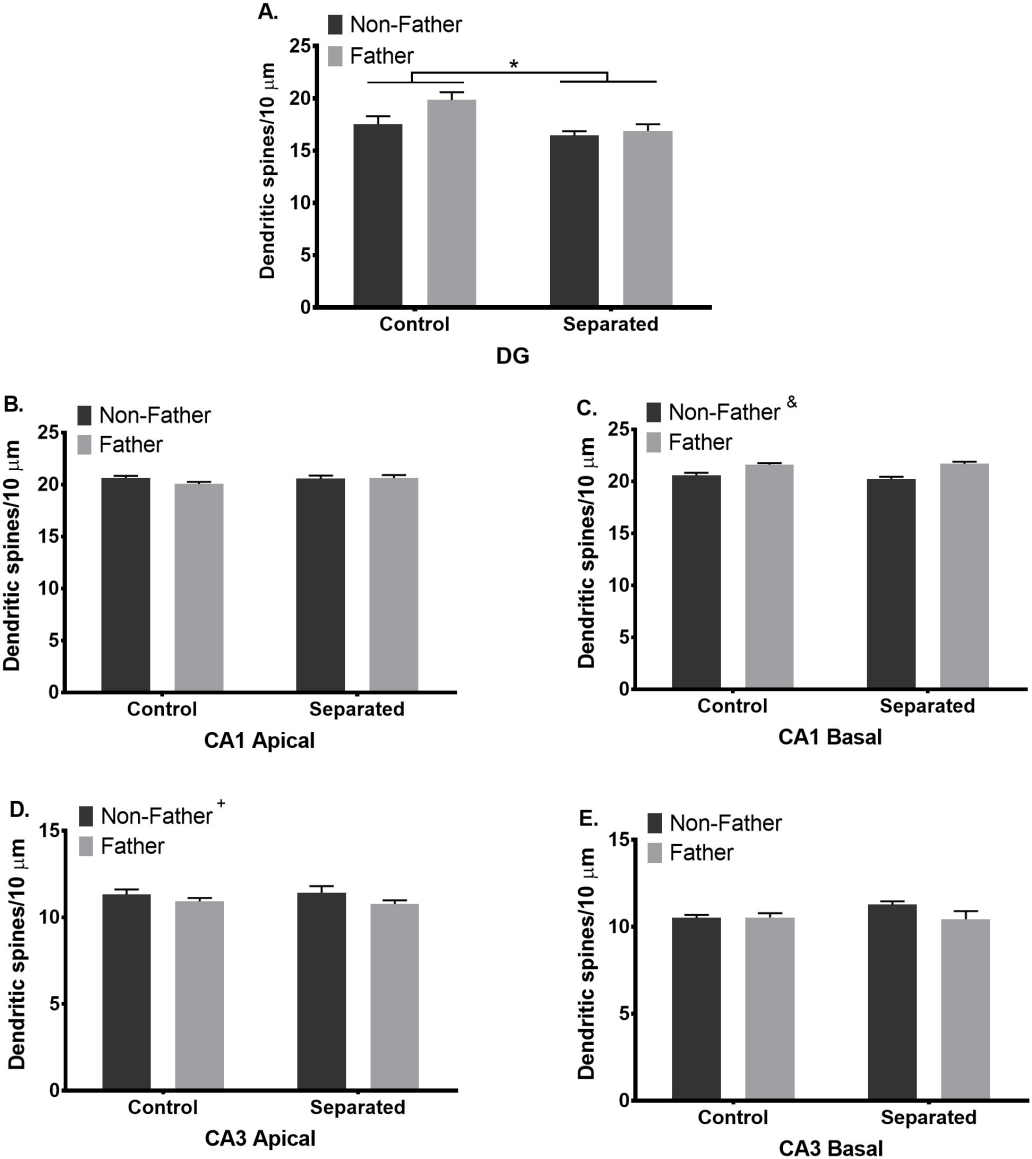
Separation and fatherhood have hippocampal subfield-specific effects on dendritic spine density in California mouse fathers. (A) Separation from mate and/or offspring decreases dendritic spine density of dentate gyrus (DG) granule cells, compared to control males. *p ≤ 0.05. (B) Neither paternal experience nor separation alters dendritic spine density of CA1 apical pyramidal cells. (C) Paternal experience increases spine density on CA1 basal dendrites, but (D) decreases spine density of CA3 apical dendrites ^+^p ≤ 0.05, non-fathers compared to fathers, ^&^p ≤ 0.001, non-fathers compared to fathers. (E) Neither paternal experience nor separation alters dendritic spine density of CA3 basal pyramidal cells. Bars represent Mean + SEM.

DG. In DG granule cells, a main effect of separation (F_(1, 30)_ = 7.429; p = 0.011; d = 0.979, η^2^ = 0.248), but not paternal experience, was observed in dendritic spine density. Overall, separation reduced dendritic spine density of DG granule cells regardless of paternal experience (Fig 2A; Table 1). While no interaction between separation and paternal experience was observed, we hypothesized that the effects of separation would be greater in fathers than non-fathers. Post-hoc analysis revealed that DG spine density was reduced in separated fathers, compared to control fathers (p = 0.009; [34]). However, this difference was not statistically significant after FDR correction was applied (q = 0.011).

**Table 1:** Separation and paternal experience alters dendritic spine density in areas CA1, CA3, and the DG of the hippocampus in California mice.

Hippocampal Subregion	Non-Fathers		Fathers		p-value		
	Control	Separated	Control	Separated	Separation	Paternal Experience	Interaction
DG	17.54 ± 0.76	16.45 ± 0.41	19.85 ± 0.74	16.89 ± 0.64	**0.011**	0.074	0.219
CA1 Apical	20.62 ± 0.22	20.55 ± 0.30	20.05 ± 0.20	20.61 ± 0.30	0.351	0.322	0.236
CA1 Basal	20.57 ± 0.26	20.23 ± 0.21	21.61 ± 0.14	21.68 ± 0.21	0.506	**0.001**	0.326
CA3 Apical	11.33 ± 0.29	11.43 ± 0.37	10.93 ± 0.18	10.78 ± 0.21	0.905	**0.049**	0.622
CA3 Basal	10.52 ± 0.16	11.27 ± 0.19	10.53 ± 0.25	10.44 ± 0.46	0.294	0.190	0.183

DG = dentate gyrus. Mean ± SEM is reported. Significant effects are in bold text.

CA1. In CA1 apical dendrites, no main effect of separation, paternal experience, or interaction was observed in dendritic spine density (Fig 2B; Table 1). However, in CA1 basal dendritic spine density, a main effect of paternal experience (F_(1, 30)_ = 36.43; p = 0.001; d = 2.168, η^2^ = 1.215), but not separation was observed. Regardless of housing condition, fatherhood increased dendritic spine density of CA1 basal dendrites (Fig 2C; Table 1). No interaction was observed.

CA3. In CA3 apical dendrites, a main effect of paternal experience (F_(1, 30)_ = 4.204; p = 0.049; d = 9.69, η^2^ = 0.140), but not separation was observed in dendritic spine density. No interaction was observed. Overall, paternal experience decreased dendritic spine density on CA3 apical pyramidal cells regardless of separation (Fig 2D; Table 1). In CA3 basal dendrites, no main effects of separation, paternal experience, or an interaction between the two were observed (Fig 2E; Table 1).

#### Dendritic tree length and branching

*DG*: Length of DG granule cell dendritic trees was not altered by separation or paternal experience, and no interaction was observed. Additionally, number of branch points on DG granule cells was not altered by separation or paternal experience, and no interaction was observed. Overall, DG granule cell complexity was not altered by fatherhood or separation (Table 2).

**Table 2:** Separation and paternal experience alter dendritic tree complexity in CA1, but not DG or CA3, of the hippocampus in California mice.

Dendritic Measures		Non-Fathers		Fathers		p-value		
		Control	Separated	Control	Separated	Separation	Paternal Experience	Interaction
**Length (μm)**	DG	802.7 ± 65.79	771.10 ± 50.93	747.64 ± 32.37	856.41 ± 62.73	0.474	0.778	0.197
	CA1 Apical	841.3 ± 51.08	1004.89 ± 48.94	637.98 ± 55.78	910.85 ± 76.62	**0.002**	**0.025**	0.394
	CA1 Basal	697.54 ± 50.80	892.8 ± 65.03	672.82 ± 72.33	864.68 ± 77.72	**0.013**	0.722	0.982
	CA3 Apical	815.68 ± 65.60	755.06 ± 30.29	758.45 ± 52.65	765.96 ± 52.09	0.646	0.689	0.556
	CA3 Basal	939.26 ± 94.52	870.56 ± 65.94	1050.86 ± 72.22	911.38 ± 92.28	0.238	0.385	0.685
**Branch Points**	DG	5.50 ± 0.41	5.33 ± 0.34	5.27 ± 0.19	5.10 ± 0.25	0.575	0.434	0.999
	CA1 Apical	8.18 ± 0.28	10.13 ± 0.51	6.03 ± 0.66	9.05 ± 0.60	**0.000**	**0.013**	0.393
	CA1 Basal	7.20 ± 0.64	8.47 ± 0.81	5.78 ± 0.66	8.40 ± 0.50	**0.009**	0.292	0.337
	CA3 Apical	6.63 ± 0.62	7.33 ± 0.45	6.82 ± 0.44	7.22 ± 0.64	0.328	0.941	0.791
	CA3 Basal	7.75 ± 0.75	6.10 ± 0.68	8.30 ± 0.53	7.48 ± 0.88	0.098	0.194	0.573

DG = dentate gyrus. Mean ± SEM is reported. Significant effects are in bold text. Shaded cells represent significant group differences following false discovery rate analysis.

**Fig 3:**
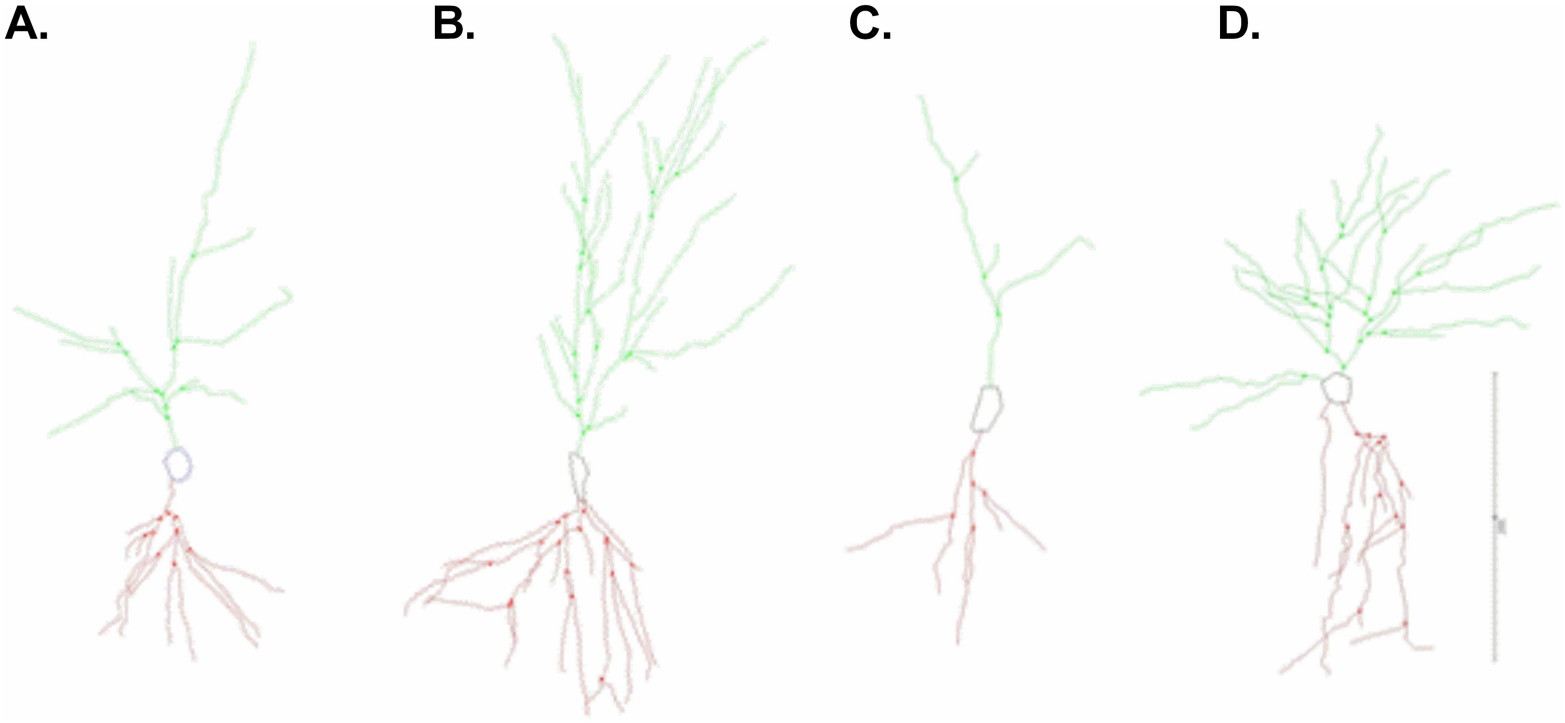
Microlucida tracings of Golgi-impregnated CA1 pyramidal cells. Representative tracings of a (A) control non-father, (B) control father, (C) separated non-father, and (D) separated father. Scale bar = 200 μm.

*CA1 apical*: On apical dendrites, the length of CA1 trees was altered by separation (F_(1, 30)_ = 11.93; p = 0.002; d = 1.241, η^2^ = 0.398) and paternal experience (F_(1, 30)_ = 5.544; p = 0.025; d = 0.846, η^2^ = 0.185) [34]. Separation increased, while fatherhood decreased, dendritic tree length of CA1 apical trees (Table 2; Fig 3). While no interaction was observed, we hypothesized that the effects of separation would be exacerbated in fathers compared to non-fathers. Post-hoc analysis revealed that separated fathers had significantly longer apical dendritic trees than control fathers (p = 0.004), however, this effect was no longer significant following FDR correction (q = 0.008). Branch points on apical dendrites were altered by separation (F_(1, 30)_ = 16.64; p = 0.000; d = 1.465, η^2^ = 0.555) and paternal experience (F_(1, 30)_ = 6.992; p = 0.013; d = 0.95, η^2^ = 0.233) [34]. As observed in dendritic tree length, separation increased, while fatherhood decreased, number of branch points on CA1 apical trees (Table 2; Fig 3). While no interaction was observed, we expected separation to have a greater effect in fathers than non-fathers. Post-hoc analysis revealed that separated fathers had significantly more branch points along apical dendrites than control fathers (p = 0.001, q = 0.004).

*CA1 basal*. Total length of CA1 basal trees was effected by separation (F_(1, 30)_ = 6.914, p = 0.013, d = 1.881, η^2^ = 0.230) but not paternal experience. Separation increased the length of CA1 basal dendritic trees (Table 2; Fig 3). No interaction was observed. Number of branch points was also altered by separation (F_(1, 30)_ = 7.876; p = 0.009; d = 1.008, η^2^ = 0.262) but not paternal experience. Separation increased the number of branch points on basal dendritic tree of CA1 pyramidal cells (Table 2). No interaction was observed, however, we expected separation to alter branch points of fathers more than non-fathers. Post-hoc analysis indicated that separated fathers had more branch points along the basal dendritic tree compared to control fathers (p = 0.013), however, this difference was no longer significant after FDR correction was applied (q = 0.010).

*CA3 apical*: Length of apical dendritic trees in CA3 was not altered by separation or paternal experience, and no interaction was observed. Number of branch points on the apical dendritic tree in CA3 was not altered by separation or paternal experience and no interaction was observed. Overall, neither separation nor fatherhood altered dendritic complexity of CA3 apical dendrites (Table 2).

*CA3 basa*l. Length of basal dendritic trees in CA3 was not affected by separation or paternal experience, and no interaction was observed. Number of branch points on CA3 basal dendrites was not affected by separation or paternal experience, and no interaction was observed. Overall, CA3 basal dendrites were not altered by separation or fatherhood (Table 2).

## Discussion

To our knowledge, this is the first study to experimentally determine the effects of mate and offspring separation on dendritic morphology within the hippocampus of a monogamous and paternal rodent. Here, we demonstrate that while separation from a mate increases passive stress-coping behaviors in male California mice, separation from offspring can magnify this effect. Additionally, our data indicate that the effects of separation and paternal experience on neuronal complexity within the hippocampus are long lasting.

In adult California mice, pair bonds are formed and maintained for life [41–44]. This species is also biparental, therefore the male provides substantial care to offspring [45]. It is likely that a strong bond exists between California mouse fathers and offspring; however, this has yet to be experimentally demonstrated. Nonetheless, this model system provides us the opportunity to examine the effects of bond, or mate, separation on affective behavior in males. As previously demonstrated in other social rodent species, bond separation increases passive stress-coping behaviors in the forced swim task [46–48]—a task that assesses behavioral despair in rodents [38]. Male California mice that were separated from either their mate, or mate plus offspring, experienced shorter latencies to immobility, longer durations of immobility, and more bouts of immobility. Furthermore, in fathers separated from their offspring on PND1, latency to the first bout of immobility was shorter and the number of immobility bouts was higher 20 days later compared to control fathers.

These data are the first to make two important observations in the monogamous and biparental California mouse. First, separation from a mate results in an impaired coping response during the forced swim test. There is a rich body of literature highlighting the relationship between bond disruption and passive stress-coping behavior [5, 7, 46]. For example, in the socially monogamous prairie vole (*Microtus ochragaster*), short and long separations from a bonded mate result in extended durations of immobility during the forced swim task in males (5 days; [46]) and females (4–6 weeks; [7, 47]). Male Siberian dwarf hamsters (*Phodopus sungorus*) are less active, have increased body mass, seminal vesicle mass, and testicular mass, and also display heightened cortisol concentrations following four weeks of separation from their bonded female [48]. Following separation, both male and female prairie voles exhibit reduced sucrose intake, which is a measure of negative affect [47]. Females also display increased immobility in the forced swim task, decreased time spent on the open arms of the elevated plus maze, and increased pup-directed attack behavior [5]. Our current findings, coupled with the existing literature, suggest that forced termination of strong pair bonds has long lasting effects on behavioral measures of coping and despair.

Second, and shown here for the first time, preventing interactions with offspring results in increased passive stress-coping behaviors in fathers—an effect that is similarly observed in maternal rodents. Disruption to mother-offspring interaction results in emotional dysregulation and negative affect [26, 27, 49]. Given these similarities, it is possible that similar mechanisms in mothers and fathers, like modulated hypothalamic-pituitary-adrenal (HPA) axis activity, may contribute to negative affect following separation from offspring. Rat dams that have been separated from their pups for 4 or 24 hours on PND 7 [27] or have experienced repeated, extended separations (180 minutes of separation from their pups per day for three weeks) [26] exhibit passive coping behavior in the FST. Temporary and repeated separations from offspring, and its effects on passive stress-coping behavior has not been assessed in paternal males of any species. Importantly, the California mouse fathers in this study were behaviorally assessed 20 days following just one day of caring for offspring. These data suggest that separation from offspring has long-lasting effects on California mouse fathers and highlights the possible strength of the father-offspring relationship.

Given the importance of the hippocampus in modulating affective behaviors [50–52] and its sensitivity to experience-induced modifications to neuronal structures [53], we examined to what extent separation and paternal experience could modulate dendritic morphology in hippocampal subregions. While paternal experience did not significantly alter DG dendritic spine density in the present study, we have previously observed this in California mouse fathers at the time of weaning [34]. Similarly, maternal rodents display increased dendritic spine density within the DG of the hippocampus [18]. These data in paternal and maternal rodents would suggest that the effects of parental experience on DG structural plasticity are similar. However, this is not always observed. While adult neurogenesis, the birth of new neurons, remains suppressed at offspring weaning in the DG of California mouse fathers and mothers [11], cell survival has returned to baseline levels in postpartum rat dams [13, 54]. Clearly, the effects of parental experience on DG structural plasticity are not uniform and may respond in species- and/or sex-dependent ways.

We also demonstrated that 4–5 weeks of separation reduced dendritic spine density in the DG of male California mice, regardless of paternal experience. Socially isolating male rats, at weaning, for eight weeks results in decreased basal dendritic length and spine density in area CA1 [55]. CA1 apical dendritic spine density is impaired in isolate-housed adult male rats compared to pair-housed males or group-housed males reared in a complex spatial environment [56]. These data suggest that separation from mate and/or offspring for 21 days can have long-lasting impairments on dendritic spine density in various hippocampal subregions.

While other measures of dendritic complexity in the DG were not altered by paternal experience or separation, separation did increase the total length and number of branch points of pyramidal cells in CA1, regardless of paternal experience or whether apical or basal dendrites were assessed. Social isolation can have many divergent effects on brain plasticity. California mouse males and females experience increased adult neurogenesis following isolation [57]. Female prairie voles, on the other hand, show decreased adult neurogenesis after six weeks of isolation [7]. After 8–9 weeks of isolation, male rats show region-specific changes in dendritic morphology [58]. Specifically, pyramidal neurons in the medial prefrontal cortex exhibit reduced branch points and dendritic spine density following isolation, but total dendritic length is increased. Additionally, the basolateral amygdala has increased dendritic arborization, decreased total length, but no change in dendritic spine density following isolation housing. Finally, the nucleus accumbens core has reduced overall dendritic length following isolation, but dendritic spine density is unchanged. Interestingly, the density of nubbin (N type) spines is greater in the visual cortex of isolate-housed aged rats compared to those living in a social environment. This is irrespective of cortical layer or dendritic segment [59]. We did not observe differences in dendritic spine density as a result of separation; however, classification of spine type was not performed in the current study. This type of analysis may reveal spine type-specific changes in density and should be investigated in future studies.

Parental experience significantly modifies pyramidal cells within area CA1 of the hippocampus. We observed increased spine density on CA1 basal, but not apical, dendrites—an observation we have previously reported [34]. However, dendritic spine density is enhanced on both apical and basal dendrites during the postpartum period in rat dams [19]. It is not clear why dendritic spine density of apical dendrites is not altered in paternal males. It is likely that species, and even sex differences, between rat dams and California mice at the end of the postpartum period influence these alterations in spine density. Changes in dendritic spine density in California mouse mothers have not been explored.

Paternal experience resulted in a retraction of dendritic arbors and a reduction in the number of bifurcations along the dendritic tree of apical dendrites in CA1. Since separated fathers remained with their offspring for only 1 day, this suggests that siring a litter is sufficient to promote long lasting effects on the complexity of CA1 apical dendrites. However, the complexity of basal dendritic trees in CA1 was not modified by paternal experience, as no differences in total dendritic length or number of branch points was observed. Therefore, the observed enhancement in dendritic spine density of CA1 basal dendrites was a result of a greater number of dendritic spines.

Paternal experience, but not separation, altered dendritic spine density of CA3 pyramidal neurons. Regardless of housing condition, fatherhood decreased dendritic spine density of apical, but not basal, dendrites. Neither housing nor paternal experience altered dendritic length or number of bifurcations. Therefore, these results indicate that fatherhood reduces the number of dendritic spines on CA3 apical dendrites. The CA3 region of the hippocampus is highly susceptible to stress [60–62] and chronic stress decreases dendritic complexity within this region in male rats [60, 61, 63–65]. Rat dams demonstrate atrophy of CA3 apical and basal dendritic trees during the postpartum period, but dendritic spine density is unaltered [19]—again highlighting the possible sex differences in parenting-induced neural morphology. Sex differences in CA3 dendritic plasticity may be a direct result of sex-specific steroid hormones [63]. Unlike males, treating postpartum female rats with corticosterone results in increased density of mushroom spines in the CA3 region of the hippocampus [66]. Currently, the mechanisms underlying sex differences in parenting-induced CA3 dendritic plasticity are unknown. It is possible that alterations in stress responsivity following offspring interaction may be sex-specific.

## Conclusion

The formation and maintenance of bonds are essential for highly social species. Our data suggest that forced dissolution of the pair bond induces passive stress-coping behaviors and contributes to region-specific alterations in hippocampal structure in California mouse males. Permanent disruption of the interactions between California mouse fathers and their offspring may further contribute to the changes in hippocampal structure and function that accompany pair bond dissolution. To our knowledge this is the first study to investigate to what extent limiting father-offspring interaction, in a biparental species, alters behavioral and neural endpoints in the father. Offspring interaction is important for emotional health during the postpartum period of males [67], a finding that has also been observed in postpartum females [26, 27, 49]. The expression of postpartum depression and anxiety in fathers can negatively impact the father-child relationship, suggesting that father-offspring interactions are important for the health of the father and can have significant effects on development of the child [68, 69]. It will be important to identify mechanisms underlying both the neural and behavioral alterations observed during the postpartum period in males and how they may be similar or different than what is observed in postpartum females. This will ultimately contribute to our understanding of parenting-induced neuroplasticity.
